# Effect of Kangfuxin Liquid enema combined with mesalazine on gestational outcomes and quality of life in child-bearing female with active ulcerative colitis: A protocol for randomized, double-blind, controlled trial

**DOI:** 10.1097/MD.0000000000023915

**Published:** 2021-02-05

**Authors:** Tong Wang, Hua Lu, Fangyuan Li, Qi Zhang

**Affiliations:** a College of Clinical Medicine, Chengdu University of Traditional Chinese Medicine; b Hospital of Chengdu University of Traditional Chinese Medicine, Chengdu, Sichuan Province, P.R. China.

**Keywords:** Kangfuxin Liquid, pregnancy outcome, randomized controlled trial, traditional Chinese medicine, ulcerative colitis

## Abstract

**Background:**

In recent years, the incidence of ulcerative colitis (UC) is on the rise, and most of them are young adults. As the peak of the disease overlaps with the childbearing age, it has a great impact on the fertility of female patients. We, therefore, conduct a randomized and controlled trial to evaluate the efficacy and safety of mesalazine enteric-coated tablets combined with Kangfuxin Liquid (KFX) enema for the child-bearing period female with active UC.

**Methods:**

In this randomized controlled study, a total of 236 eligible patients will be assigned to the experimental group (n = 118) or the control group (n = 118) in a 1:1 ratio. The control group will be taken mesalazine enteric-coated tablets combined with placebo enema and the experimental group will be taken mesalazine enteric-coated tablets combined with KFX enema. Participants will receive 8 weeks of intervention treatment and 3 months of maintenance treatment before pregnancy. The primary assessment is the Mayo score. Secondary outcomes include mucosal healing, faecal calprotectin (FC), Inflammatory Bowel Disease Quality (IBDQ), and pregnancy outcome.

**Discussion:**

This study will provide evidence regarding the efficacy and safety of KFX enema used before pregnancy on halting active UC, reducing the relapse rate during pregnancy, improving pregnancy outcome, and the quality of life.

**Trial registration:**

Chinese Clinical Trials Register identifier, ChiCTR2000039161, registered on October 20, 2020.

## 1 Introduction

Ulcerative colitis (UC) is one of the two inflammatory bowel diseases (IBD) characterized by bloody diarrhea, abdominal pain, and mucus stools. According to a systematic review,^[1]^ Europe has the highest annual incidence of UC—24.3 per 100,000 people. Although the number in Asia and the Middle East is only 6.3 per 100,000 people, in these countries such as Japan and China, the incidence of UC has been increasing remarkably.^[2,3]^ Not only the intestinal symptoms of UC itself but also its impact on all aspects of the daily life of patients, especially females, have received considerable critical attention. Of particular concern is that UC occurs most frequently in the young female with the highest incidence peak in the second or third decades of life^[4]^ which is the essential prime reproductive age for women.

In recent years, there has been an increasing amount of study on the impact of the female with UC on pregnancy and neonatal. Many pieces of evidence show that pregnant women with UC have a higher incidence rate of abnormal pregnancy and delivery, such as the delivery of premature, low birth weight infants, and venous thromboembolism (VTE) risk in both pregnancy and the postpartum period than those without UC, and there are significant differences in active UC.^[5–7]^ Furthermore, female with UC increased the risk of infant birth defects overall. In a retrospective cohort study^[8]^ of 7798 cases of UC in Quebec, Canada between 1989 and 2016, UC was associated with 1.53 times the risk of central nervous system defects. Another study^[9]^ concluded that neonatal are at an elevated risk of limb and urinary defects. The problem about the damages of UC medication to their unborn children or heredity increases their fertility concerns.^[10]^ Guideline^[10]^ recommends that women plan to get pregnant when in remission. However, it cannot be ignored that an increased risk for UC relapse during pregnancy^[11]^ Similarly, a meta-analysis^[12]^ by Abhyankar et al has demonstrated that the recurrence rate and severity of UC influenced by disease activity at the time of conception. In addition, several lines of evidence suggest that UC has an interactive relationship with Anxiety and Depression (A&D). Individuals with UC are at increased risk of A&D. Meanwhile, UC with comorbid A&D are more challenging to manage. The selective protective effect of antidepressant treatment on UC confirms that it has a two-way relationship with A&D^[13,14]^ A prospective cross-sectional study^[15]^ in the United States or Denmark suggest that activity UC significantly correlated with sexual dysfunction. Besides, it exacerbates dysmenorrhea and gastrointestinal symptoms during menstruation.^[16]^ Overall, for females with UC, there are unique challenges in a different decade of life, spanning from menstruation, sexual health, mental health to fertility, and pregnancy. Consequently, It is essential for the patients to have optimal management of UC prior to and during pregnancy to achieve favorable maternal and neonatal outcomes, and preferable quality of life.^[17]^

Mesalazine, a first-line treatment for mild to moderate UC, is recommended to continue to be used during pregnancy to reduce the significant increase of relapse rate.^[17]^ It is classified as Class B by the Food and drug administration which is safe and tolerable.^[18]^ However, there are still 23.6% to 36.5% of patients who have undergone standard treatment with mesalazine with unsatisfactory mucosal healing (endoscopy score ≥ 2) and even worsening of the symptom during the 12 months follow-up.^[19,20]^

As integrative therapy or alternative medicine, traditional Chinese medicine (TCM) is safe and economical in improving symptoms. In recent decades, magnanimous evidence from clinicians suggests that TCM has some beneficial effect on UC.^[21–23]^ Kangfuxin Liquid (KFX) is an extract of Periplaneta americana dried worms, which has been approved by the National Medical Products Administration (Z51021834). Periplaneta americana is an insect of the cockroach family, recorded in Shen Nong Ben Cao Jing more than 2000 years ago in China, included many active substances such as polyols and peptide which presented the bioactive functions of anti-inflammation and promoting mucosal barriers repair.^[24]^ Periplaneta americana has been diffusely applied in China against various diseases. Modern research indicates that the Periplaneta americana prevents inflammatory responses to protects against renal and hepatic fibrosis, accelerates mucosal healing by increasing the expression of EGF and VEGF in granulation tissueim.^[25–27]^ Over the past decade, it has been emphasized the effect of Periplaneta americana on the gastrointestinal ulcer. It is now well established that Periplaneta americana is effective and safe for gastrointestinal ulcer patients from a variety of studies including clinical trials, animal experiments, and system analysis.^[28–32]^ However, KFX treatment before pregnancy has not been evaluated in detail in terms of improving or maintaining the progression of UC from before, during pregnancy to childbirth. We designed an RCT to evaluate the effects of mesalazine combined with KFX enema before pregnancy on halting active UC, reducing the relapse rate during pregnancy, improving pregnancy outcome, and the quality of life.

## 2 Methods and analysis

### 2.1 Study design

**Figure 1:**
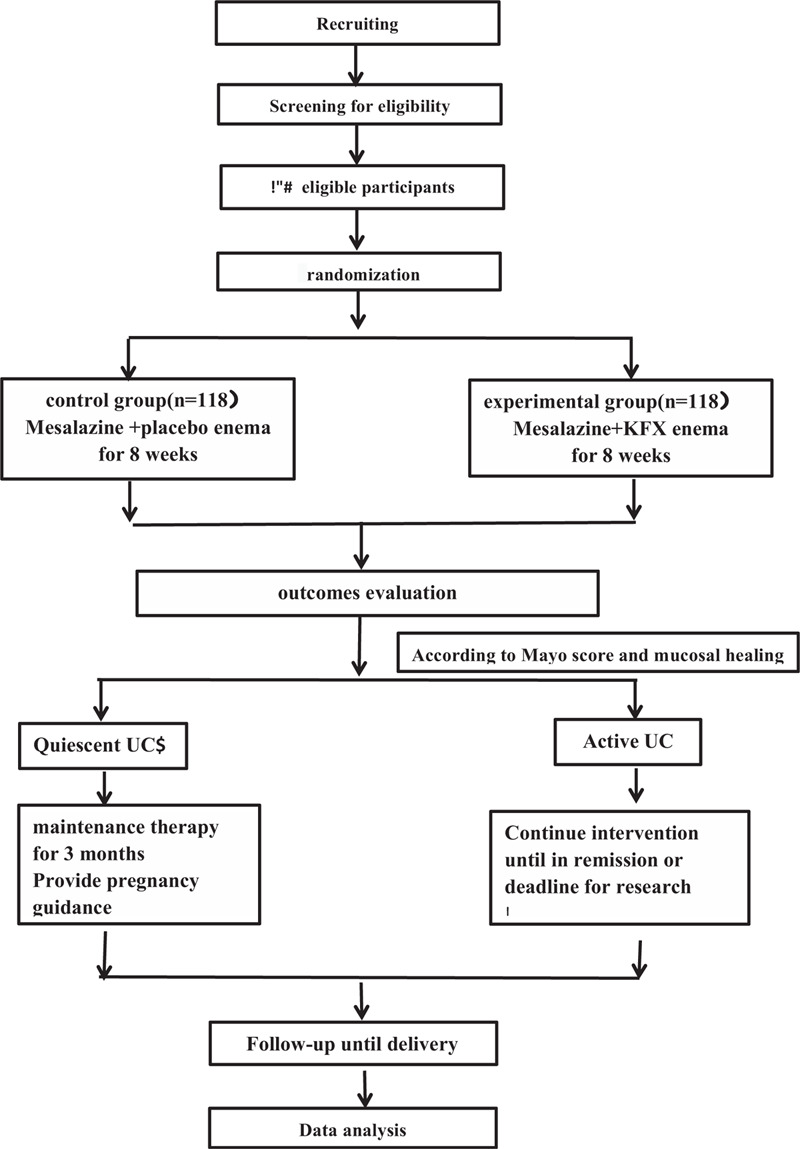
Trial flow chart.

This study is designed as a randomized, controlled, double-blind trial. With mesalazine enteric-coated tablets as the control, to explore the efficacy of mesalazine combined with KFX enema in the treatment of active UC in women with fertility requirements and to improve pregnancy outcome and quality of life, reduce the recurrence rate. Prospective participants will be asked to talk face to face with the researcher for a baseline screening visit after diagnosis. After the 8 weeks of treatment, they will be followed for another 3 months of maintenance therapy. This study will start in October 2020 and continue until April 2022. The study schedule is detailed in Table 1. The flowchart of the trial is shown in Figure 1.

**Table 1:** Data collection points.

	Screening	Randomization	treatment	Follow-up (maintenance therapy)	Follow-up (pregnancy until delivery)		
Visits	①	②	③	④	⑤	⑥	⑦
Time point	4 weeks before treatment	Day 0	Weeks 1–8	Weeks 9–20	Months 0–3	Months 4–6	Months 7 to delivery
Age	X						
Duration of disease	X						
Disease history	X						
Comorbidity	X						
Clinical examination	X						
control group			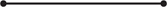				
experimental group			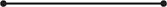				
Mayo score	X		X	X			
Mucosal healing	X		X	X			
IBDQ	X		X	X	X	X	X
Faecal calprotectin	X		X	X	X	X	X
pregnancy outcome					X	X	X
Adverse events			X	X	X	X	X
Causes of dropout			X	X	X	X	X
Data analysis							X

### 2.2 Ethic approval

The research protocol is conducted in accordance with the principles of the Declaration of Helsinki^[33]^ and has been approved by the Sichuan Regional Ethics Review Committee on Traditional Chinese Medicine (approval no. 2019KL-072). Also, this trial has been registered in the Chinese Clinical Trial Registry (Registration No. ChiCTR2000039161).

### 2.3 Participants

A total of 236 women in the child-bearing period with UC will be recruited after signed the informed consent. They will be invited to the Department of gynecology, Teaching Hospital of Chengdu University of Traditional Chinese Medicine. The printed recruitment posters will be post in the hospital to recruit participants. All participants should meet the diagnostic criteria according to the Chinese consensus on the diagnosis and treatment of IBD.^[34]^ Eligible participants will be implemented randomly with a 1:1 allocation ratio in the control group with (n = 118) or the experimental group (n = 118).

### 2.4 Inclusion criteria

Patients who meet all of the following conditions will be considered for enrollment. The inclusion criteria are as follows:

1. Females, aged between 20 and 45 years;
2. Patients who meet the criteria of active UC refer to Chinese consensus on diagnosis and treatment;
3. Patients preparing for pregnancy;
4. Patients who willing to participate in the study, sign an informed consent and can be followed up

### 2.5 Exclusion criteria

The exclusion criteria are as follows:

1. Patients with infertility caused by diseases of reproductive system organs or male infertility
2. Patients with intestinal organic diseases and other serious complications such as local stenosis, intestinal obstruction, intestinal perforation, colon cancer, colorectal cancer, bacillary dysentery, Amoebic dysentery, chronic schistosomiasis, radiation enteritis, etc.
3. Patients with the allergic constitution or allergic to any known medicine components of Mesalazine or KFX
4. Patients with serious diseases such as liver, kidney, hematopoietic system and mental disorder

### 2.6 Sample size

The sample size was determined by the primary study results. According to previous reports, the effective rate of the experimental group based on the Mayo, score ranges from 57.5% to 76.47%,^[35,36]^ and the average rate is about 66.99%. The effective rate of the control group ranges from 41.25% to 55.65%,^[22,37]^ and the average rate is about 48.45%. We will use the following formula to estimate sample size:

$${\text{n}}_{\text{c}}=\frac{[\pi_{t}(1-\pi_{t})/k+\pi_{c}(1-\pi_{c})]{\left(u_{1-\partial /2}+u_{1-\beta }\right)}^{2}}{{\left(\pi_{t}-\pi_{c}\right)}^{2}}$$

With a 5% significance level (α = 0.05), 80% Power (β = 0.2), and 10% dropout rate, at least 118 patients should be enrolled in each group and 236 total participants will be recruited.

### 2.7 Randomization and blinding

Participants will be randomly allocated according to a random list of numbers generated with SPSS21.0 software. Opaque envelopes containing an allocation sequence number will be used by an independent staff who needs to prepare the assignments and conceal the allocation sequence. Blinding will be strictly maintained to intervention staffs, participants, the statistician, and staff who obtained outcome measurement. They will not know the corresponding relations between numbers and different groups. By using an identical liquid placebo in color, consistency, and smell to the KFX, blinding will be ensured.

## 3 Interventions

In the experimental group, mesalazine enteric-coated tablets (supplied by LOSAN Pharma GmbH 0.5 g/tablet) will be taken orally 1.0 g per time and 3 times a day for 8 weeks continuously. At the same time, the KFX (supplied by Sichuan Good doctor Panxi Pharmaceuticals) will be given retention enema treatment 100 mL per time, 1 time a day. KFX need to be heated to 37°C and poured into a one-time enema bag. The enema tube will be inserted into the anus for about 10 cm. The control group enteric-coated mesalazine tablets will be taken orally 1.0 g per time and 3 times a day for 8 weeks. The enema method of placebo is the same as that of the treatment group. During the treatment period, patients in both groups avoid irritant and crude fiber food and supplemented with an adequate amount of folic acid (2 mg/d). Eight weeks later, according to the guidelines,^[17]^ UC patients in remission (Mayo score ≤2 and normal intestinal mucosa or no active inflammatory) will continue to be treated with maintenance therapy Maintenance therapy is based on the treatment mentioned above, but the dose is reduced (mesalazine 0.5 g/time, 3 times/day, KFX/placebo enema 50 mL) for 3 months. Subsequently, the patient in stable condition will be given active pregnancy guidance. Both groups of patients change to oral mesalazine (0.5 g/time, 3 times/day) from pregnancy to delivery. Due to a lack of research on the safety of KFC being used during pregnancy, KFX will be discontinued once patients in remission enter the active pregnancy preparations stage. All drugs should be the same lot number. In the course of the study, it is forbidden to add other drugs or interventions that may interfere with the trial. Cases in which symptoms have not alleviated or even aggravated, the participants voluntarily withdrew, and no medication or any follow-up records due to poor compliance will be regarded as excluded cases, and participants will be required to complete the assessments as much as possible.

## 4 Outcome measures

### 4.1 Primary outcome

The primary outcome in this trial is the Mayo score at baseline, after an 8-week intervention, and 3-month remission period maintenance therapy.

The Mayo score scoring standard is based on references^[38]^ (Table 2). A total score of <2 is divided into symptom relief, 3 to 5 is divided into mild activity, 6 to 10 is divided into moderate activity, and 11 to 12 is divided into severe activity.

**Table 2:** Mayo scoring system for assessment of ulcerative colitis activity.

Score	Stool frequency A	Bloody stools B	Colonoscopy	Physician's global assessment C
0	Norma (no. of stools for this patient)	No blood seen	Normal or inactive disease	Normal
1	1–2 times more than normal	Streak of blood with stool less than half the time	Mild disease (erythema, decreased vascular pattern, mild friability)	Mild
2	3–4 times more than normal	Obvious blood with stool most of the time	Moderate disease (marked erythema, lack of vascular pattern, friability, erosions)	Moderate
3	5 or more times more than normal	Blood alone passes	Severe disease (spontaneous bleeding, ulceration)	Severe

### 4.2 Secondary outcomes

The secondary outcomes include:

1. pregnancy outcome
  Pregnancy outcome include pregnancy rate (%), abnormal pregnancy rate (%), and live birth rate (%). In this study, abnormal pregnancy is defined as any of the following: abortion, threatened abortion, premature delivery, delivery of a low-birth-weight infant, VTE, and congenital defect, etc—any pregnancy-related event causing illness in the mother or fetus.
2. mucosal healing rate (%)
  Patients with remission UC can be seen as normal colon mucosal appearance, pseudopolyp formation, or scar-like changes.
3. IBDQ
  The patients’ quality of life is evaluated by the IBDQ^[39]^ included the 32 items and divided into 4 modules: bowel symptoms, systemic symptoms, emotional function, and social function. Each question has graded responses from 1 to 7, and thus scored from 32 to 224 points, with a higher score indicates a better quality of life.
4. FC
  Endoscopies as the “gold standard” determine the presence of mucosal inflammation in the bowel. Some studies propose that endoscopy should be used with caution during pregnancy to avoid adverse pregnancy outcomes.^[40,41]^ Therefore, for the noninvasive monitoring of the activity or relapse of UC, the FC will be measured to replace endoscopyat after pregnancy. The concentration of FC has a strong correlation with the level of intestinal inflammatory activity during pregnancy.^[42]^

### 4.3 Safety outcomes

KFX has been used clinically for many years and has satisfactory safety profiles. To monitor the adverse reactions, Safety evaluation will include electrocardiogram, routine examination, blood routine test, hepatic function, renal function, urine routine test, and stool routine test. All safety results will be evaluated at baseline, 8th, 20th week after the intervention, 0th, 4th, 7th month, and until delivery after pregnancy.

If any adverse event occurs including toxicity and side effects, researchers should record it in the case record form (CRF) in detail and provide a proper treatment emergently. A serious adverse event will be reported to the ethical committee within 24 h. In addition, the patient will be determined whether to terminate the trial based on the severity.

### 4.4 Quality control and trial monitoring

In order to maintain the quality of the study, all researchers will accept specialized training including how to select and exclude participants, how to correctly manipulate intervention measures, how to fill the case report forms uniformly, how to evaluate outcomes, and manage data. The trial data of the patient will be recorded in the case record and then input into the spreadsheet respectively by 2 data managers. Once the CRF is completed, the data will be locked and not to be allowed to modify. To guarantee the objectivity of the data, the data managers submit it to the statistician for statistical analysis blinded during the trial.

### 4.5 Statistical analysis

The statistician performs Statistical analyses of data by using SPSS21.0 software (International Business Machines Corp., Armonk, NY). Intention-to-treat principle approach for efficacy and safety analysis will be used. As dealing with the missing values, multiple imputations method will be applied. Continuous variables with normal distribution, expressed as Mean ± Standard Deviation, will be analyzed by *t* test. In contrast, nonparametric tests will be adopted when variables are kept in the abnormal distribution. Categorical data presented by frequency and percentage will be analyzed by the chi-square test or Fisher exact test. All the tests will be conducted two-sided, and a *P*-value < .05 will be considered as the significant level.

## 5 Discussion

Up to now, the etiology of UC has not been fully elucidated. Genetics, environmental factors, autoimmunity, and gut microbiota are generally considered to be key risk factors for UC. Research by analyzing the intestinal bacterial community indicates that UC patients have intestinal dysbiosis with reduction of biodiversity,^[43]^ In particular, Firmicutes and Bacteroidetes known to promote gut health have been reduced most significantly.^[44]^ Moreover, the impaired epithelial barrier is caused by key pro-inflammatory cytokines (tumor necrosis factor-α (TNF-α, interferon (IFN)-γ, interleukin (IL)-13, etc)is a pathogenic factor for UC.^[45,46]^ In recent years, Mitochondriopathy is considered to be the pathogenic process in UC, loss of mitochondrial homeostasis brings about defective energy production, increased mitochondrial oxidative stress, and the release of pro-inflammatory DAMPs1 molecular.^[47–50]^

Generous studies based on UC mouse models trials show that Periplaneta americana increases the Lactobacillales in UC mouse, decreases *E coli* intestinal bacteria translocation, modulate the flora structure, restore the intestinal-immune system, stimulate fibroblasts proliferation and accumulate collagen significantly to promote the colonic morphology and mucosal barrier recovery.^[30–32,51]^ Simultaneously, Periplaneta americana improves the accumulation of myeloperoxidase (malondialdehyde, superoxide dismutase, catalase, and nitric oxide), reduce inflammatory factor expression (TNF-a, IL-6, MMP-9, and MMP-2 ), and block the MAPK/NF-κB signaling pathway.^[29]^ Currently, high-quality research and evidence are lacking in KFX for the female with fertility requirements. Therefore, we conduct a randomized and controlled trial to evaluate the efficacy and safety of mesalazine combined with KFX enema before pregnancy in the treatment of active UC in women with fertility requirements and to improve pregnancy outcome and quality of life, reduce the recurrence rate. In particular, we use the FC instead of endoscopic examination which is not be suggested to be used to assess the symptom progression of the UC during the pregnancy. We hope the results clarify the value of KFX as the adjunctive therapy for a child-bearing period female with active UC.

### 5.1 Trial status

The recruitment of patients started on October 30, 2020, and it is expected that by January 30, 2021, the required sample size will be reached. This protocol was submitted before completion of recruitment.

## Acknowledgments

We would like to thank all the patients who will participate in the trial and the staff for their support.

## Author contributions

**Conceptualization:** Tong Wang, Hua Lu.

**Investigation:** Fangyuan Li, Qi Zhang.

**Supervision:** Hua Lu.

**Writing – original draft:** Tong Wang.

**Writing – review & editing:** Tong Wang.
